# Supplementary material for: Around one third of current Arctic Ocean primary production sustained by rivers and coastal erosion
Source: Nat Commun. 2021 Jan 8;12:169. doi: 10.1038/s41467-020-20470-z (PMC7794587; doi:10.1038/s41467-020-20470-z)
Supplement: Supplementary file 1 — Supplementary Information [file 41467_2020_20470_MOESM1_ESM.pdf]

Supplementary Information for “Around one third of current Arctic Ocean primary production sustained by rivers and coastal erosion” by Terhaar et al.

Supplementary Online Material: Around one third of current Arctic Ocean primary production sustained by rivers and coastal erosion

Jens Terhaar[1,2,3,4*], Ronny Lauerwald[1,2,5], Pierre Regnier[2], Nicolas Gruber[6], Laurent Bopp[7]

[1] Laboratoire des Sciences du Climat et de l'Environnement, LSCE/IPSL, CEA-CNRS-UVSQ, Université Paris-Saclay, 91191 Gif-sur-Yvette, France

[2] Biogeochemistry and Earth System Modelling, Department of Geoscience, Environment and Society, Université Libre de Bruxelles, Belgium

[3] Climate and Environmental Physics, Physics Institute, University of Bern, Switzerland

[4] Oeschger Center for Climate Change Research, University of Bern, Switzerland

[5] Université Paris-Saclay, INRAE, AgroParisTech, UMR ECOSYS, 78850, Thiverval-Grignon, France

[6] Environmental Physics, Institute of Biogeochemistry and Pollutant Dynamics, ETH Zurich, Zurich, Switzerland

[7] LMD/IPSL, Ecole Normale Supérieure/PSL University, CNRS, Ecole Polytechnique, Sorbonne Université, Paris, France

***Jens Terhaar**

Climate and Environmental Physics, Physics Institute

University of Bern

Sidlerstrasse 5

3012 Bern

Switzerland

jens.terhaar@climate.unibe.ch

Supplementary Table 1. Catchment properties used in regressions and their source.

Parameter	Data sources
Carbonate Index for lithology	(Dürr et al., 2005)
Areal extend of continuous permafrost	(Brown et al., 1998)
Glacial cover	World Glacier Inventory (http://nsidc.org/data/g01130.html).
Annual runoff (for regression equation)	Average observed runoff from Argo data
Lake cover	Global Lake and wetland data base (Lehner and Döll, 2004)
Topsoil organic carbon content (Topsoil C_{org})	Harmonized World Soil Data base (HWSD)
Topsoil clay content	(Nachtergaele et al., 2010)

Supplementary Table 2. Results for the multiple-linear regressions for annual fluxes and concentrations.

Predicted Variable	Intercept	Predictors							R^2	RMSE
		Carbonate index [%]	Perma-frost [%]	Glacial cover [%]	Runoff [cm yr-^{1}]	Lake cover [%]	Topsoil C_{org} [%]	Topsoil Clay [%]		
Inorganic carbon fluxes										
C_T[1] [g C $m^{-2}y^{r-1}$]	1.513	0.059	-0.014	2.207	0.046				0.92	0.3681
Alkalinity [g $CaCO_3$ $m^{-2}yr^{-1}$]	4.491	0.280	-0.061	8.358	0.221				0.93	1.373
Concentrations of DOC and nutrients										
DOC[2] [mg C L^{-1}]	17.143		-0.050			-1.375	1.142	-0.544	0.92	0.54
DON[3] [ug N L^{-1}]	442.922		-1.668			-34.171	21.609	-11.72	0.88	18.94
DIN[4] [ug N L^{-1}]	65.030		-0.869			-16.840	12.850	3.865	0.96	9.78
P_T[5] [ug P L^{-1}]	19.330		-0.211			-5.941	5.647	-0.171	0.94	2.67
Si [mg Si L^{-1}]	3.660	-0.027	-0.009	0.217		-0.144			0.86	0.22

[1]Total dissolved inorganic carbon
[2]Total dissolved organic carbon
[3]Total dissolved organic nitrogen
[4]Total dissolved inorganic nitrogen
[5]Total dissolved phosphorus

Supplementary Table 3. Minimum and maximum concentration based on observed annual fluxes from ArcticGRO[9,18] used to define the extrapolation corridor. All values in mg L^{-1}. Alkalinity is in mg $CaCO_3$ L^{-1}.

Species	Min	Max
C_T	6.82	22.54
Alkalinity	21.0	84.7
DOC	4.36	10.79
DON	0.10	0.29
DIN	0.05	0.22
TDP	0.008	0.046
Si	1.84	4.12

Supplementary Table 4. Parameters for the statistical model of seasonality in fluvial matter fluxes (Eqs. 1 &2). The RMSE is unitless like the predicted variables, which give the proportion of the monthly flux relative to the average annual flux.

	a_1	a_2	b_1	b_2	RMSE
C_T	1.074	0.6221			0.1950
Alkalinity	1.0678	0.8023			0.2141
DOC	0.79379	1.23824			0.4301
DON	0.83105	1.18158			0.4696
DIN	1.02323	0.7842	0.49375	0.67591	0.4356
TDP	0.85018	1.12083			0.3833
Si	1.03644	0.69412			0.3149

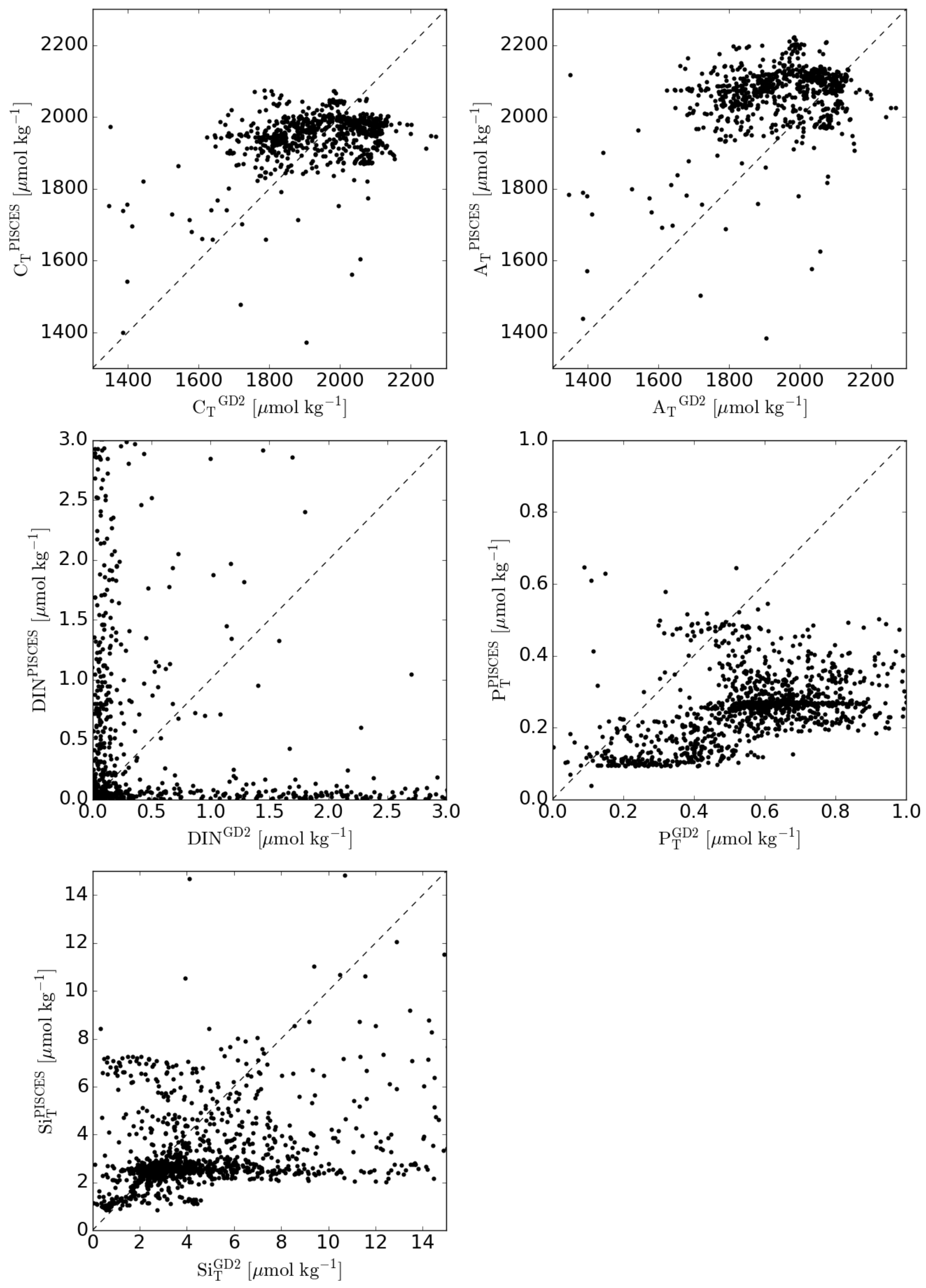

Supplementary Figure 1. Comparison of observed vs. simulated carbon and nutrient concentrations at the Arctic Ocean surface. Observed concentrations in July and August from GLODAPv2 (Olsen et al., 2016) and 6-year monthly averages (2005-2010) of simulated concentrations at the same location and for the same month.

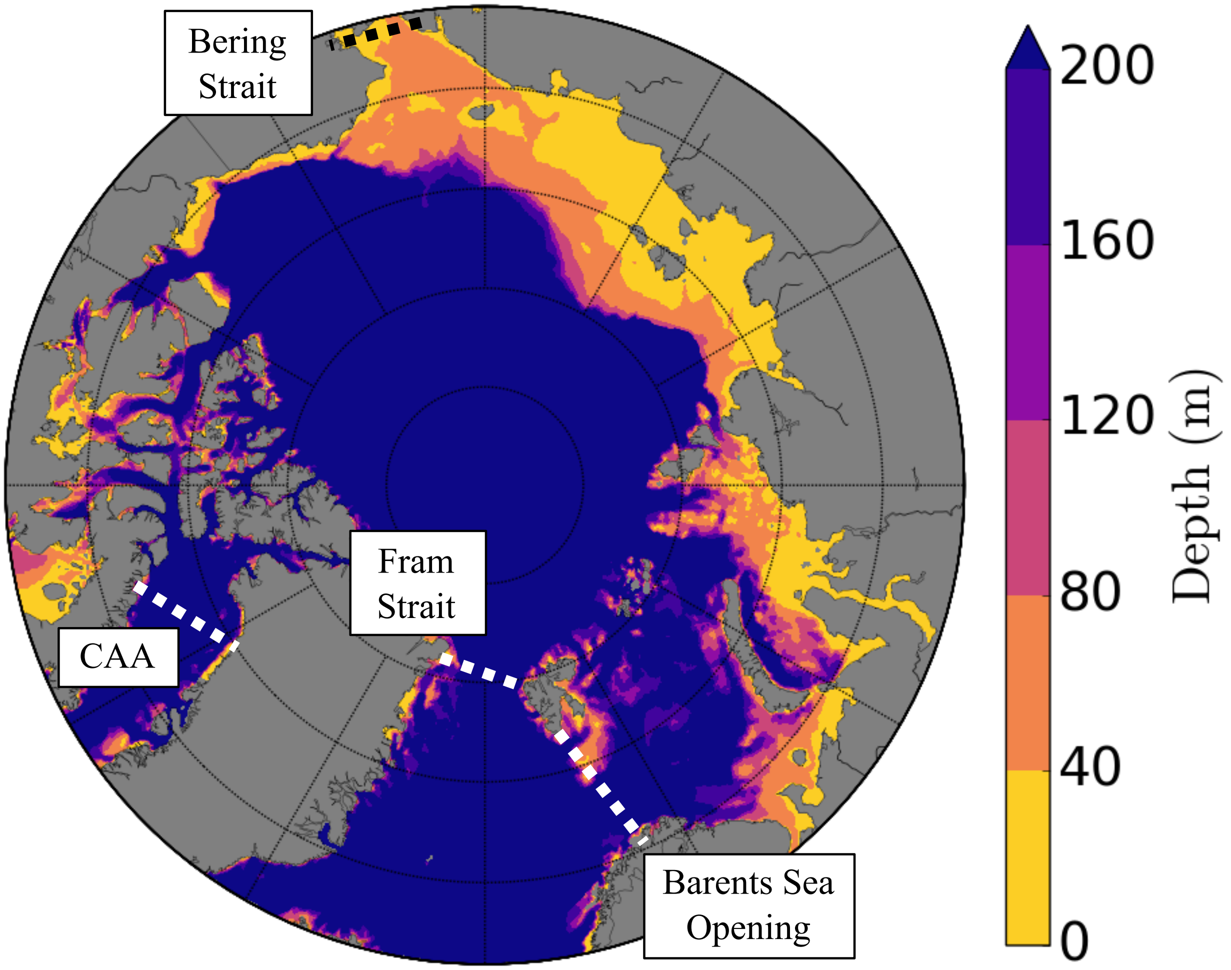

Supplementary Figure 2. Bathymetry of the Arctic Ocean shelf seas. The boundaries of the Arctic Ocean as defined in this study are marked by dashed lines.

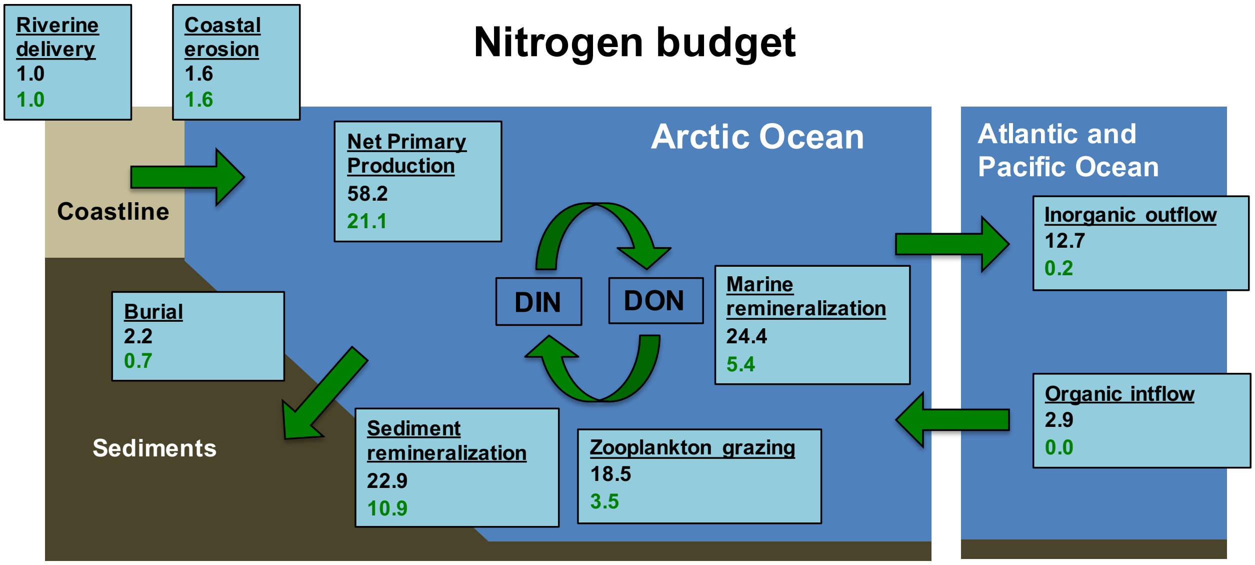

Supplementary Figure 3. Simulated Arctic Ocean nitrogen budget. The budget includes nitrogen exchange with adjacent oceans, import from rivers and coastal erosion and export via burial. Within the Arctic Ocean nitrogen is constantly transformed from its inorganic to its organic form via NPP and from its organic to its inorganic form via marine remineralisation of dead organic matter, remineralisation in sediments, and grazing of phytoplankton by zooplankton followed by excretion of inorganic matter. Black numbers indicate the total rates and green numbers the rates that are exclusively driven by terrigenous nitrogen input. Note that the budget is off balance as the total Arctic Ocean nitrogen inventory decreases by 9.4 Tg N yr^{-1} over the simulation period whereas the inventory of terrigenous nutrients increases by by 1.7 Tg N yr^{-1}.

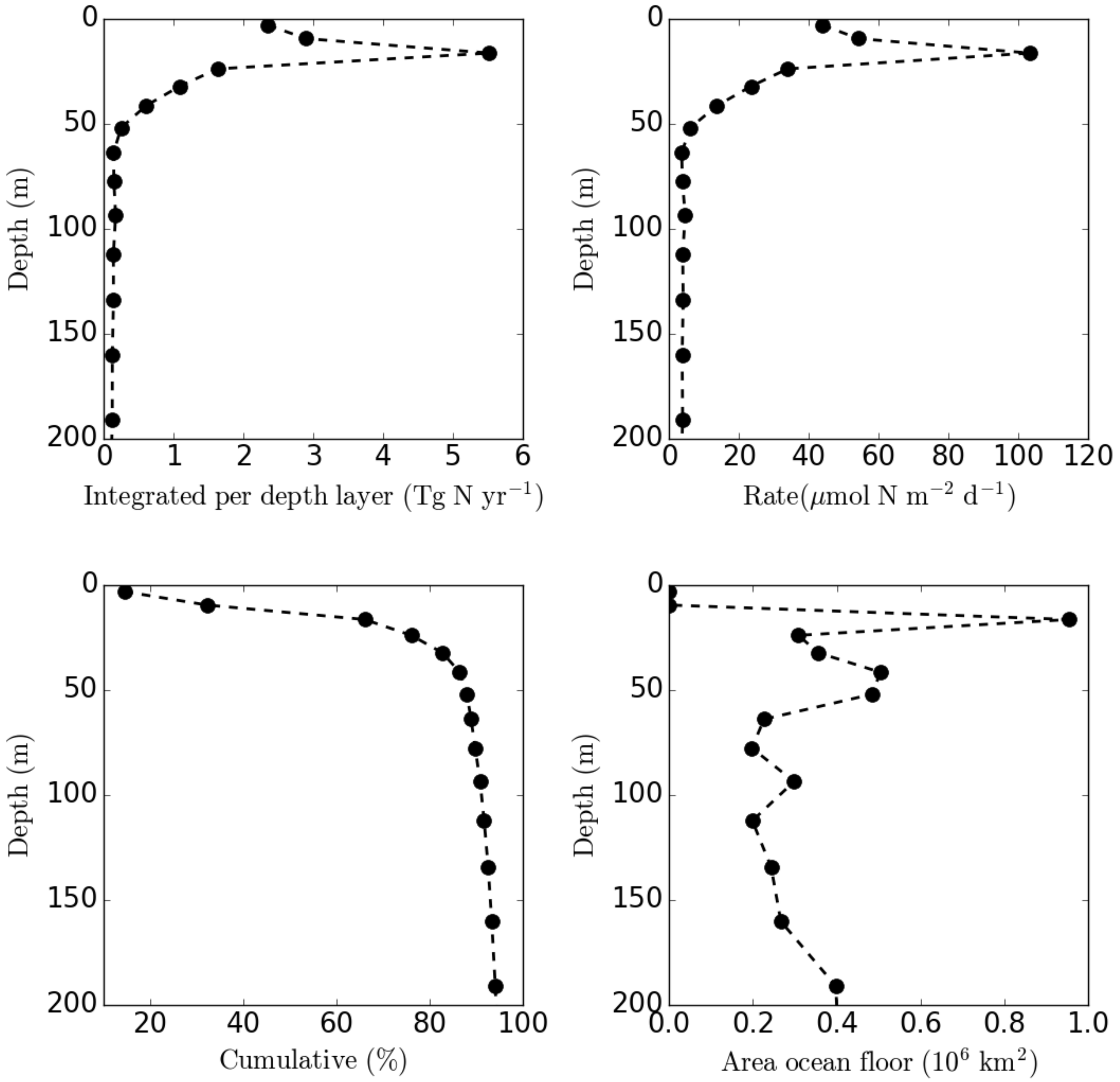

Supplementary Figure 4. Vertical profiles of simulated organic nitrogen remineralisation in the water and sediments. a) integrated remineralisation rate per depth layer, **b)** rate of remineralisation per depth layer, **c)** cumulative remineralisation (from 0% at the surface and 100% at the ocean floor), and **d)** ocean floor area per water depth in the Arctic Ocean.

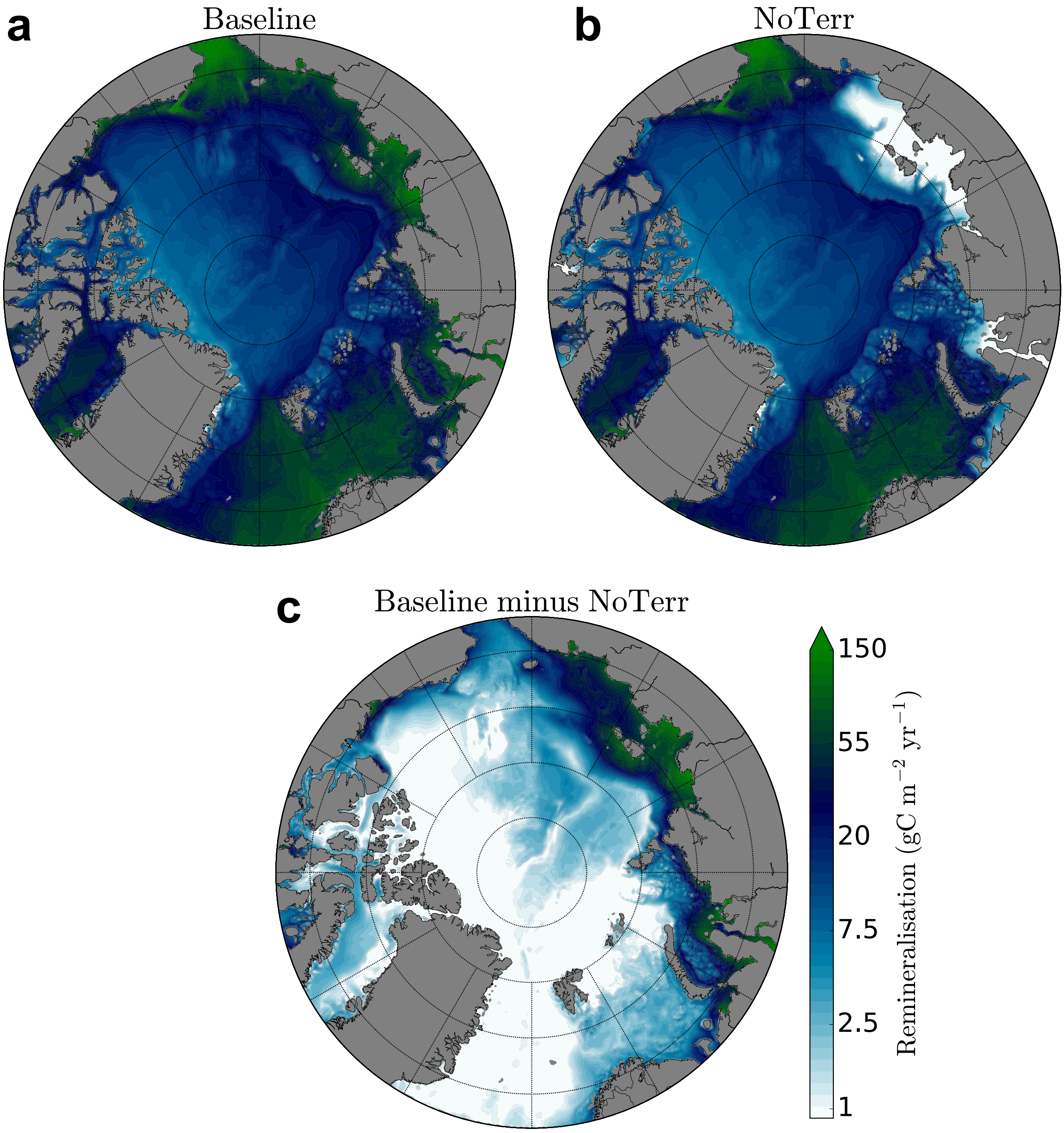

Supplementary Figure 5. Annual Arctic Ocean organic matter remineralisation. a, simulated Arctic Ocean organic matter remineralisation with observation-based nutrient input from rivers and coastal erosion (Baseline) **b,** simulated Arctic Ocean organic matter remineralisation without input of terrigenous nitrogen (NoTerr) and **c,** the difference between simulated Arctic Ocean NPP in **(a)** Baseline and **(b)** NoTerr simulations.

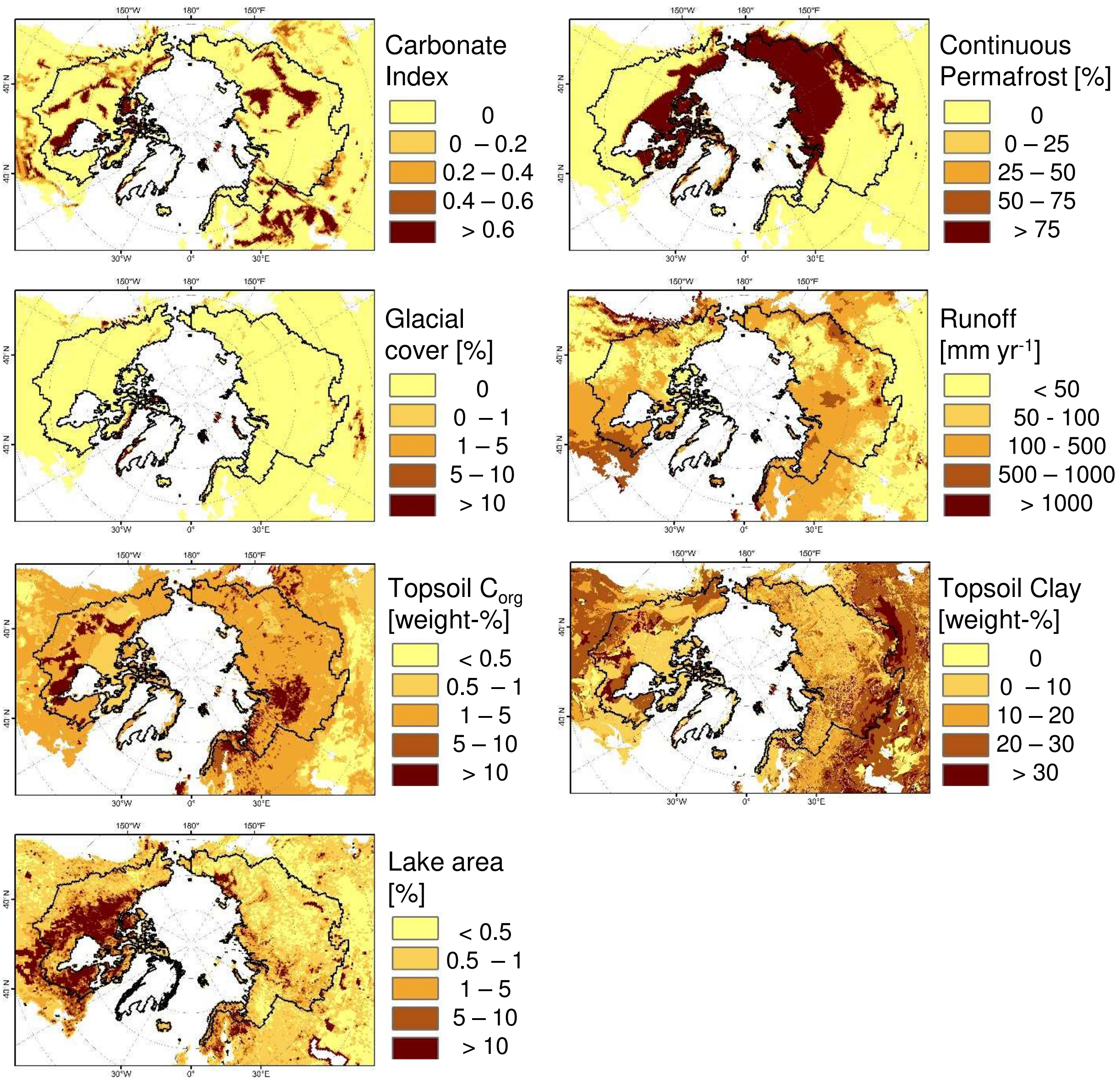

Supplementary Figure 6. Maps of river catchment properties. Catchment properties used in regressions as listed in Table S1. Greenland was largely taken out of these spatial predictions (white color) because there is no significant runoff coming from there. Only some small coastal watersheds are taken into account.

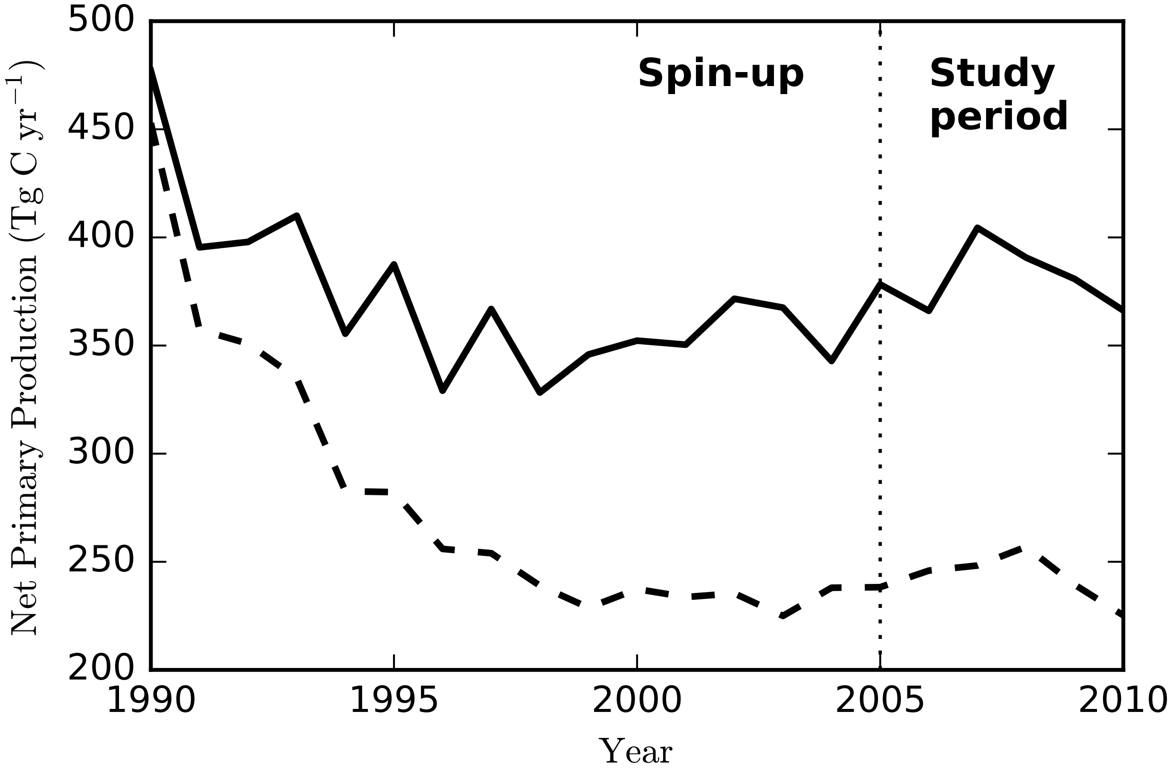

Supplementary Figure 7. Time-series of annual Arctic Ocean net primary production. Annual Arctic Ocean net primary production over the 21 years simulation period with external supply of terrigenous nutrients (solid line) and without (dashed line). The dotted line indicates the end of the spin-up and the beginning of the study period in 2005.

Supplementary references

Brown, J., O. Ferrians, J. A. Heginbottom, and E. Melnikov. 2002. Circum-Arctic Map of Permafrost and Ground-Ice Conditions. Version 2. Boulder, Colorado USA: National Snow and Ice Data Center. [Date Accessed]. doi:10.3133/cp45

Dürr, H. H., Meybeck, M., Durr, S. H., Dürr, H. H. and Dürr, S. H.: Lithologic composition of the Earth's continental surfaces derived from a new digital map emphasizing riverine material transfer, Global Biogeochem. Cycles, 19(4), doi:10.1029/2005gb002515, 2005.

Lehner, B. and Döll, P.: Development and validation of a global database of lakes, reservoirs and wetlands, J. Hydrol., 296(1–4), 1–22, doi:10.1016/j.jhydrol.2004.03.028, 2004.

Nachtergaele, F., Velthuizen, H. van, Verelst, L., Batjes, N. H., Dijkshoorn, K., Engelen, V. W. P. van, Fischer, G., Jones, A. and Montanarela, L.: The Harmonized World Soil Database, Proc. 19th World Congr. Soil Sci. Soil Solut. a Chang. World, Brisbane, Aust. 1-6 August 2010, 34–37, doi:3123, 2010.

Olsen, A., Key, R. M., van Heuven, S., Lauvset, S. K., Velo, A., Lin, X., Schirnick, C., Kozyr, A., Tanhua, T., Hoppema, M., Jutterström, S., Steinfeldt, R., Jeansson, E., Ishii, M., Pérez, F. F., and Suzuki, T.: The Global Ocean Data Analysis Project version 2 (GLODAPv2) – an internally consistent data product for the world ocean, Earth Syst. Sci. Data, 8, 297–323, https://doi.org/10.5194/essd-8-297-2016, 2016.
